# Ensemble Empirical Mode Decomposition Analysis of EEG Data Collected during a Contour Integration Task

**DOI:** 10.1371/journal.pone.0119489

**Published:** 2015-04-24

**Authors:** Karema Al-Subari, Saad Al-Baddai, Ana Maria Tomé, Gregor Volberg, Rainer Hammwöhner, Elmar W. Lang

**Affiliations:** 1 Department of Biology, Institute of Biophysics, University of Regensburg, Regensburg, Germany; 2 Department of Linguistics, Literature and Culture, Institute of Information Science, University of Regensburg, Regensburg, Germany; 3 Department of Electrical Engineering, Telecommunication and Informatics, Institut of Electrical Engineering and Electronics, Universidade de Aveiro, Aveiro, Portugal; 4 Department of Psychology, Pedagogics and Sport, Institute of Experimental Psychology, University of Regensburg, Regensburg, Germany; University of Salamanca- Institute for Neuroscience of Castille and Leon and Medical School, SPAIN

## Abstract

We discuss a data-driven analysis of EEG data recorded during a combined EEG/fMRI study of visual processing during a contour integration task. The analysis is based on an ensemble empirical mode decomposition (EEMD) and discusses characteristic features of event related modes (ERMs) resulting from the decomposition. We identify clear differences in certain ERMs in response to contour vs noncontour Gabor stimuli mainly for response amplitudes peaking around 100 [*ms*] (called *P*100) and 200 [*ms*] (called *N*200) after stimulus onset, respectively. We observe early *P*100 and *N*200 responses at electrodes located in the occipital area of the brain, while late *P*100 and *N*200 responses appear at electrodes located in frontal brain areas. Signals at electrodes in central brain areas show bimodal early/late response signatures in certain ERMs. Head topographies clearly localize statistically significant response differences to both stimulus conditions. Our findings provide an independent proof of recent models which suggest that contour integration depends on distributed network activity within the brain.

## Introduction

The mammalian visual system is able to recognize a multitude of objects within a visual scene. Object recognition presupposes the ability for contour integration and figure—ground separation. Visual integration is defined as the processes of combining the output of neurons, which carry small attributes of a scene, into a complex structure more suitable for the guidance of behavior. Contour integration is one of the most elementary tasks during visual feature integration. Still it is debated whether contour integration is confined to the visual cortex or whether higher brain areas are involved as well. The traditional theory of visual processing suggests a hierarchy of serial processing steps through a bottom-up cascade of discrete visual areas [1]. But this strict bottom—up processing is challenged by more recent theories proposing a parallel bottom-up and top-down information flow [2]. The ability to integrate oriented contrast edges (Gabor elements) into a contour depends on the spacing and orientation of the Gabor elements relative to the path orientation [3, 4]. Similar principles apply in the multi-stable organization of regular arrays of elements in rows and columns [5, 6]. Other, more general, stimulus properties also seem to influence the binding of contour elements: Closed contours are more easily detected than open ones [7, 8]. Likewise, symmetric contours are also easier to detect than asymmetric ones [9]. Indeed, contour integration improves when Gabor elements are oriented perpendicular to the contour within a closed area, and deteriorates, when these elements are oriented parallel to the contour [10].

### Event—related potentials and contour integration

Analyzing brain activities during visual processing is largely based on non-invasive techniques like *functional Magnetic Resonance Imaging* (fMRI) and/or *Electroencephalography* (EEG). The former offers good *spatial resolution*[11], while the latter excels in *temporal resolution* hence finds applications also in brain—computer interfacing [12, 13]. Traditionally, EEGs are studied at the level of event related potentials (ERPs) which represent averages over a sufficiently large number of single trial recordings. Characteristic ERP components and their related latencies are then compared for different stimulus conditions. Several studies investigate differences between contour and non-contour stimulus conditions for various components of event related potentials (ERPs) [14, 15, 16, 17]. Such differences arise mostly for mean peak amplitudes of the ERP components *P*1 and *N*1 denoting the first positive and first negative components appearing after stimulus onset, respectively. Early studies already demonstrated that modulations during contour integration do not only vary with context but also with task demand [14]. This study already shows that contour integration involves a neural network including anterior and posterior brain areas in addition to the visual cortex. Recently, electrophysiological studies [15] provided further clear evidence that context modulates the electrophysiological signature of contour integration at early stages of visual processing. Modulating effects were mainly seen for the ERP components *N*1 and *P*2, corresponding to the first negative and the second positive ERP components appearing after stimulus onset. However, no effect was seen for the first positive ERP component *P*1. In summary, context primarily exerts a modulatory effect on the *N*1 component. These studies thus highlight the dynamic interplay between perceptual grouping and the context in which it operates. A more recent contour integration study using EEG analysis [16] advocated the hypothesis that perceptual grouping involves top-down selection rather than a mere pooling of afferent information streams encoding local elements in the visual field. Differential brain activity, i. e. statistically significant differences in stimulus response amplitudes for the ERP component *N*1, occurring roughly at a latency of 170–180 [*ms*], could be detected only during a contour integration task within parietal, lateral occipital and primary visual areas. If the contour stimuli were presented with a concurrent task (i.e., if the contours were not the detection target), then no differences in brain activity were found between contour and non-contour stimuli. The study concludes that contour integration seems to be based on selecting information from primary visual areas, and appears to be controlled by the lateral occipital cortex. This conclusion corroborates results of another recent EEG study on contour integration [18]. A contour and a non-contour stimulus were presented within the same trial in fast succession, with the task to indicate whether the contour was shown within the first or within the second presentation interval. As a result, differences in brain activity between contour and non-contour stimuli occurred for stimuli shown in the first interval, but were completely absent for stimuli shown in the second interval. Thus, top-down information obtained from the serial presentation shaped the brain activity in response to contour stimuli.

The above mentioned debate about competing theories concerning the mechanisms of contour integration has been the focus of yet another electrophysiological study. Tackling the question whether serial facilitative interactions between collinear cells in the primary visual cortex (V1) or pooling of inputs in higher-order visual areas determine contour integration, the authors applied high-density electrophysiological recordings to assess the spatio-temporal dynamics of brain activity in response to Gabor contour stimuli embedded in Gabor noise versus control stimuli. The study concluded that the earliest effects could be observed in the ERP component *N*1 rather than in the component *C*1 of the visual evoked potential. Inverse modeling identified sources in the lateral occipital complex (LOC) within the ventral visual stream as the generators of *N*1 modifications. Also modulatory contextual effects appeared only at this later processing period. The authors claim that these results are consistent with a pooling of information from primary visual areas in higher cortical areas only at a latency characteristic for the occurrence of the *N*1 stimulus response component [19].

Concurrent to traditional ERP research, there is also increasing interest in oscillatory brain responses during contour integration. Oscillatory brain activity is thought to reflect rhythmic changes between relatively high and relatively low excitability within a neural population. By synchronizing neural activity, groups of neurons can be transiently linked into neural assemblies to jointly process a given task [20]. With respect to contour integration, several studies revealed local power increases in beta frequencies (15–30*Hz*) during contour compared to non-contour processing. The beta power difference occurred relatively early (< 160 *ms*) and mostly at parietal and occipital electrodes across studies [18, 21]. Furthermore, increased neural long-range synchronization has been observed during contour compared to non-contour processing within theta (4–7 Hz) [22], alpha (8–12 Hz) [18] and beta frequencies [16]. Differences in high-frequency (gamma) oscillations, sometimes assumed to be a correlate of conscious visual perception [23], have not yet been found during contour integration. The results together show that brain activity differences during contour and non-contour processing are not only reflected in the ERP amplitude, but also in neural oscillations within low-to-mid frequencies.

Technically, EEG and fMRI data sets can be recorded in separate sessions or simultaneously. Integration of both, EEG and fMRI, recordings into one dataset for combined data analysis can be performed either in a symmetrical or an asymmetrical way. The latter methods include fMRI—directed EEG analysis and EEG-directed fMRI analysis [24]. Symmetrical data fusion methods mainly resort to different variants of Independent Component Analysis (ICA). Simultaneously recording EEG and fMRI signals is a demanding technique in terms of data recording and signal processing. However, their combination can reveal both the location of active brain areas and the temporal order of their activation. A very recent example is provided by a study of the dynamics of contour integration in the human brain, where EEG and fMRI data were acquired simultaneously during passively viewing Gabor stimuli under contour and non-contour conditions. By applying JointICA to the EEG and fMRI responses of the subjects, the authors gained temporally and spatially highly resolved brain responses during contour integration which could not be derived from unimodal recordings. Within EEG recordings, they found differences for stimuli with and without contours around 240 [*ms*] after stimulus onset, in early visual (V1/V2) as well lateral occipital areas. Furthermore, they found an additional later activity, occurring roughly at a delay of 300 [*ms*], in early visual areas for less salient contours, possibly reflecting re-entrant processing of such stimuli. Another combined EEG and fMRI study revealed that contour detection depends on the information transfer between lateral occipital and parietal brain areas [22], where a good detection performance required a high functional connectivity between these sites. Together these studies indicate that contour detection is accomplished within cortical networks, involving feedback loops between higher and lower visual processing areas.

### Signal decomposition techniques

Several signal decomposition techniques are available for a more detailed data analysis. Especially artifact removal, i. e. the extraction of signal components unrelated to cognitive brain activities, using blind signal separation techniques like principal and independent component analysis (PCA, ICA) are standard techniques available in toolboxes like EEGLAB [25]. Such exploratory data analysis is hampered by the intrinsically non-stationary nature, and the non-linear couplings involved in signal generation. To help alleviate such issues, recently, an empirical nonlinear analysis tool for complex, non-stationary temporal signal variations has been pioneered by N. E. Huang et al. [26]. Such technique is commonly referred to as Empirical Mode Decomposition (EMD), and, if combined with Hilbert spectral analysis, it is called *Hilbert—Huang Transform* (HHT). EMD utilizes empirical knowledge of oscillations intrinsic to a time series in order to represent them as a superposition of components with well defined instantaneous frequencies. They adaptively and locally decompose any non-stationary signal in a sum of *Intrinsic Mode Functions* (IMF) which represent zero-mean, amplitude- and (spatial-) frequency-modulated components. EMD represents a fully data-driven, unsupervised signal decomposition which does not need any *a priori* defined basis system. EMD also satisfies the perfect reconstruction property, i.e. superimposing all extracted IMFs together with the residual slowly varying trend reconstructs the original signal without information loss or distortion. Thus EMD lacks the scaling and permutation indeterminacy familiar from blind source separation (BSS) techniques. Because EMD operates on sequences of local extremes, and the decomposition is carried out by direct extraction of the local energy associated with the intrinsic time scales of the signal itself, the method is thus similar to traditional Fourier or Wavelet decompositions. It differs from the wavelet-based multi-scale analysis, however, which characterizes the scale of a signal event using pre-specified basis functions. Owing to this feature, EMD, and even more so its noise-assisted variant called *Ensemble Empirical Mode Decomposition* (EEMD), is a highly promising signal processing technique in dealing with problems of a multi-scale nature. Note that with EMD a data representation as an expansion into intrinsic modes is generated from the signal itself and no predefined basis system, as for example in Wavelet decompositions, is used for the signal representation. Thus an EMD decomposition reflects in a natural way the characteristics of non-stationary signals in either time or spatial domains. Note further that Fourier transforms have constant frequencies and weights, while Hilbert transforms allow the frequency as well as the amplitudes to vary over time.

In a recent study [27], we explored the potential of two-dimensional ensemble empirical mode decomposition (2DEEMD) to extract characteristic textures, so-called *bidimensional intrinsic mode functions* (BIMFs), from the fMRI-related part of the current, combined EEG-fMRI data sets which where taken while performing a contour integration task. To identify most informative textures, i. e. BIMFs, a support vector machine (SVM) as well as a random forest (RF) classifier were trained for two different stimulus/response conditions. Classification performance was used to estimate the discriminative power of extracted BIMFs. The latter were then analyzed according to their spatial distribution of brain activations related with contour integration. Results distinctly show the participation of higher brain areas, most notably frontal fields, in contour integration.

Given the background disussed above, we suppose that EEMD is able to extract intrinsic signal modes, so-called event related modes (ERMs), which contain decisive information about responses to contour and non-contour stimuli. Such ERMs should appear at various electrode locations indicating the presence of extended neuronal networks which process such stimuli. We further hypothesize that such response signatures are better visible, with a high statistical significance, in these modes rather than in the original recordings. Also any latencies related to such signal components could be qunatified more precisely.

### Outline of the paper

In the present study we concentrate on electrophysiological signatures within EEG recordings of the above mentioned fMRI/EEG study. We again advocate the use of EEMD to investigate neural correlates of contour integration via intrinsic modes extracted from the EEG signals, recorded while applying two visual Gabor stimulus conditions i. e. *contour true* (CT) and *non-contour true* (NCT), and studying the related electrophysiological response signals. The manuscript is organized as follows: Section *Materials and Methods* is devoted to a description of the dataset available and the way, data has been acquired. In section *Ensemble Empirical Mode Decomposition* we provide a concise summary of an EEMD algorithm and give some necessary details for its implementation. Section *Results* accounts for details of the EEG analysis and summarizes the main findings. It presents a detailed description of the EEMD analysis applied and quantifies the results obtained. Component time courses and related head topographies further illustrate these results. The last section offers a thorough discussion of these results.

## Materials and Methods

### Subjects

The subjects participating in the study encompassed 5 male and 13 female volunteers between 20–29 years old, i. e. (22.79 ± 2.7) [*years*]. All subjects were right-handed and had normal or corrected-to-normal vision. Based on self-reports, the subjects had no neurological or psychiatric disorders, brain injuries or drug dependencies. This study also was approved by the local ethics committee (study number 10-101-0035). Subjects were treated according to the principles laid down in the Helsinki declaration.

### Gabor Stimuli

**Fig 1:**
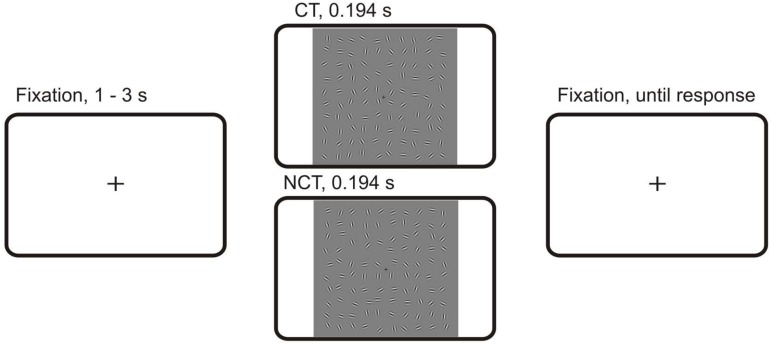
Gabor stimuli. The stimuli used in the present study.

The stimuli were generated with a procedure similar to that of [28]. Stimulus displays contained odd symmetric Gabor elements arranged in an invisible 10 by 10 grid subtending 16.6 deg×16.6 deg of visual angle. The corresponding stimulus protocol is illustrated in Fig 1. The luminance distribution *L*(*x*, *y*) of a single Gabor element is defined by the equation

$$\begin{array}{c} L\left(x,y\right)=L_{0}\left(1+s\left(x,y\right)\cdot g\left(x,y\right)\right) \end{array}$$ (1)

where *L*(*x*, *y*) [*cd*/*m* ^2^] is the luminance at point (*x*, *y*) and *L*_0_ is the background luminance. The function *s*(*x*, *y*) represents a 2D—sinusoid, describing the carrier wave, and *g*(*x*, *y*) the related Gaussian envelope, describing the amplitude modulation. These functions are given by

$$\begin{array}{c} s\left(x,y\right)=C\sin[k_{x}\cdot x\cos\left(\theta \right)+k_{y}\cdot y\sin\left(\theta \right))] \end{array}$$ (2)

where *C* = 0.9 is the Michelson contrast, ∥ **k** = (*k*_*x*_, *k*_*y*_)^*T*^ ∥ [*rad*/*m*] = 2*πf* [*cpd*] is the angular wave number with *f* = 3 [*cpd*] the corresponding spatial frequency in [*cycles*/*deg*], and *θ* is the orientation from vertical which depends on the experimental condition. Furthermore,

$$\begin{array}{c} g\left(x,y\right)=\exp(-\frac{x^{2}+y^{2}}{2\sigma^{2}}) \end{array}$$ (3)

where *σ* = 0.25 deg is the standard deviation of the Gaussian envelope, measured in degrees of visual angle.

For contour displays, a path of 10 invisible line segments was constructed and placed at a random location within the stimulus area, with the restrictions that none of the segment centers, where the Gabor elements were finally placed, fell into the inner 2×2 grid cells, and that at least 4 segment centers fell into the inner 6×6 grid cells. This ensured that the Gabor path did not cross the central fixation mark, and that the eccentricity of the path was not too large. The angle between adjacent line segments was the path angle *α* plus an orientation jitter Δ*α* drawn from a uniform distribution *p*(Δ*α*) ∈ [−1, +1]. Gabor elements were placed at the center of each line segment and aligned to the segments orientation. The separation *s* between neighbouring elements depended on the length of the corresponding line segments. It was chosen as *α* ± *δα* = 2 ± 0.55 degrees of visual angle. After setting up the Gabor path, empty grid cells were filled with randomly oriented Gabor elements. The size of the grid cells was set to $2s/(1+\sqrt{2})=1.66$ degrees of visual angle. This ensured that mean distance between distracting Gabor elements was close to the mean distance between the elements making up the Gabor path. The distracting Gabor elements were placed in the center of each grid cell and jittered vertically and horizontally by ± 0.55 degrees of visual angle. New Gabor elements were not drawn if their visible part overlapped with an already existing Gabor element by more than 5 pixels. The whole stimulus was withdrawn if more than 10 Gabor elements could not be drawn. Thus, each stimulus contained 90–100 Gabor elements. For constructing non-contour displays, the same algorithm was used as for the construction of contour displays but rotating adjoining Gabor elements by ± 45 deg. Thus, non-contour displays resembled contour displays with respect to spacing, positioning and the number of elements, but did not contain a Gabor path.

For the experiment, a set of 150 non-contour stimuli was generated, which was the same for all subjects. Then a set of 150 contour stimuli was generated separately for each subject, where the path angle *α* was adjusted to the individual maximum tolerable path angle. These angles were obtained during behavioral pre-testing and ranged from 21 deg → 34 deg.

### Experimental procedure

Subjects were positioned supine in the scanner. The visual stimuli were back projected onto a translucent circular screen (LCD video projector, JVC DLA-G20, Yokohama, Japan) and was seen on a mirror reflecting the projected image. A standard PC running Presentation 12.0 (Neurobehavioral Systems Inc., Albany, Canada) was used for stimulus presentation. The projector had a resolution of 800 by 600 pixels at a refresh rate of 72 [*Hz*]. The viewing distance to the projection screen was 64 [*cm*].

The trials started with the presentation of a central fixation cross that remained visible throughout the experimental block. After a random interval from 1000–3000 [*ms*], a stimulus was presented for 194 [*ms*], followed by a blank screen. The next trial started after the response, or after a time-out of 3000 [*ms*] if the subject did not respond. The behavioral responses were recorded with two fiber-optic response boxes (Lumitouch, Photon Control, Ltd, Burnaby, BC, Canada) where one key was provided for each finger of the left and the right hand, respectively. The subjects were instructed to detect contour stimuli. One half of the subjects used the left hand for a *contour* response and the right hand for a *non-contour* response. For the other half of the subjects the response mapping was reversed. Altogether, the subjects were presented with 300 stimuli in a random order, partitioned into 15 blocks with 20 trials each.

### Data acquisition

The EEG was recorded concurrently with fMRI in a Siemens Allegra 3 Tesla Head Scanner. In this manuscript we focus on the EEG data. The fMRI results are reported in a separate work [27].

The EEG was recorded with an MR-compatible 64 channel EEG system (BrainAmp MR plus, Brain Products, Gilching, Germany). The scalp EEG was obtained from 62 equidistant electrodes that were mounted in an elastic cap (EasyCap, Herrsching-Breitbrunn, Germany) and were referenced to FCz during recording. Impedances were kept below 20 [*k*Ω]. The signals were amplified between 0.1100 [*Hz*], with a notch filter at 50 [*Hz*] in order to cancel out mains hum. The sample rate was at the maximum resolution of 5000 [*Hz*]. To control for eye-movement artifacts, the vertical electrooculogram was recorded from an electrode placed below the left eye. An electrocardiogram (ECG) electrode was placed below the left scapula in order to facilitate the off-line removal of cardioballistic artifacts. The clock of the EEG amplifier was synchronized with the clock output of the MR scanner using a *SynchBox* manufactured by Brain Products (Gilching, Germany).

### EEG Preprocessing

MR gradient switching produces large artifacts in the EEG each time a new slice is collected. A second source of artifacts is the movement of electrodes and conductive blood inside the MR scanner due to the cardiac cycle. The continuous EEG data were cleaned from both gradient and cardioballistic artifacts by means of artifact template subtraction as implemented in the FMRIB plug-in for EEGLAB [25, 29].

- Gradient artifact onsets were determined relative to the MR volume onset marker that was recorded along with the EEG. With respect to gradient artifacts, a template was constructed for each slice artifact and for each channel separately and then subtracted from the actual artifact. The template consisted of a moving average of 21 neighboring slices and a linear combination of the major principal components describing the residual artifacts that remain after artifact subtraction. These were determined automatically by means of sorted eigenvalues. The corrected data were down—sampled to 500 [*samples*/*s*] and highpass-filtered (FIR) at 0.5 [*Hz*]. Bad stretches of data in the continuous EEG, due to incomplete gradient artifact removal or other idiographic artifacts, were identified and removed by visual inspection.
- Cardioballistic artifacts occur around heartbeats which show a characteristic deflection in the ECG electrode denoted as *QRS complex*. The FMRIB plug-in provides a reliable algorithm for the detection of *QRS onsets*. For removing cardioballistic artifacts, a temporal principal component analysis (PCA) was performed on each EEG channel. The first three components were taken as an optimal basis set for describing shape, amplitude and scale of the artifact. This set was fit to, and then subtracted from, each artifact instance. As for the removal of gradient artifacts, this was performed for each channel separately.

Preprocessed EEG data subsequently were subjected to an independent components analysis (ICA) using the extended INFOMAX algorithm [30]. Components related to artifacts were identified by visual inspection of the component topographies and power spectra. Main sources of artifacts were eye blinks, eye movements, tonic muscle activity, as well as residual pulse and gradient artifacts. Components identified as artifact-related were removed, and the remaining components were back-projected into the EEG signal space. The data was then segmented into intervals of 3 [*s*] duration, centered around the stimulus onset, and baseline-corrected within the whole interval. Single trials were again inspected and rejected if they contained artifacts. The overall rejection rate was between 6.7% and 42% across subjects (mean 22.5%), i. e., between 174–280 trials per subjects (on average 232.5 for both conditions) were available for the analysis.

EEG data were analyzed using custom code and the EEGLAB toolbox developed at the Swartz Center for Computational Neuroscience (SCCN), USA [25] (http://sccn.ucsd.edu/eeglab/). For signal analysis, the segmented data were further reduced to intervals of 1000 [*ms*] duration, each containing an onset of a Gabor stimulus. The epochs (trials) to be analyzed extended from *t*_0_−150 [*ms*] → *t*_0_+850 [*ms*] relative to stimulus onset at *t*_0_, corresponding to 500 samples.

Hence, these single trial signals are denoted as

$$\begin{array}{c} x^{\left(ch\right)}\left(t_{n},s\right)\;\text{with}\;n=-B,-B+1,\ldots 0,1,\ldots M-1,\;ch=1,2,\ldots C \end{array}$$

where *ch* denotes the index for the recording position (channel), *s* ∈ *SC* = {*CT*, *NCT*} denotes the stimulus condition and *n*_*t*_ = 1, … *N*_*t*_ with *N*_*t*_ the total number of trials. The index *t*_*n*_ is related to discrete time and *t*_0_ indicates the time of stimulus presentation, meaning that the segment starts *B* = 75 samples before stimulus onset *t*_0_ and lasts more *M* = 425 samples.

In brain studies, evoked potentials are extracted from single trial recordings by averaging single-trial signals, corresponding to a particular stimulus, over all trials. In this study, two different stimulus conditions, either contour true (CT) or non-contour true (NTC), were randomly presented. Then, for each condition, average signals $x_{avg}^{\left(ch\right)}(t_{n},s\in SC)$ were estimated

$$\begin{array}{c} \begin{array}{cc} x_{avg}^{\left(ch\right)}\left(t_{n},s\in SC\right)=\frac{1}{N_{SC}}\sum_{s\in SC}x^{\left(ch\right)}\left(t_{n},s\right) & n=-B,-B+1,\ldots ,0,\ldots ,M-1 \end{array} \end{array}$$

resulting, for each participant, in two sets of ERP signals $x_{avg}^{\left(ch\right)}(t_{n},CT)$ and $x_{avg}^{\left(ch\right)}(t_{n},NCT)$ using *N*_*CT*_ and *N*_*NCT*_ trials, respectively.

### Ensemble Empirical Mode Decomposition

Roughly a decade ago, an empirical nonlinear analysis tool for complex, non-stationary time series has been pioneered by N. E. Huang et al. [26]. It is commonly referred to as *Empirical Mode Decomposition* (EMD) and if combined with Hilbert spectral analysis it is called *Hilbert—Huang Transform* (HHT). It can be applied to any non-stationary and also nonlinear data set and represents a heuristic data decomposition technique which adaptively and locally decomposes any non-stationary time series in a sum of *Intrinsic Mode Functions* (IMF) which represent zero-mean amplitude and frequency modulated components. The EMD represents a fully data-driven, unsupervised signal decomposition and does not need any *a priori* defined basis system. EMD also assures perfect reconstruction, i.e. superimposing all extracted IMFs together with the residual trend reconstructs the original signal without information loss or distortion. However, if only partial reconstruction is intended, it is not based on any optimality criterion rather on a binary *include* or *not include* decision. The empirical nature of EMD offers the advantage over other signal decomposition techniques like *Exploratory Matrix Factorization* (EMF) [31] of not being constrained by conditions which often only apply approximately. Especially with biological signal processing, one often has only a rough idea about the underlying modes or component images, and frequently their number is unknown [32, 33]. In addition, perfect reconstruction is hampered by intrinsic scaling indeterminacies.

Eventually, the original signal *x*(*t*) can be expressed as

$$\begin{array}{ccc} x(t) & = & \sum_{j}c^{\left(j\right)}\left(t\right)+r\left(t\right)\\ c^{\left(j\right)}\left(t\right) & = & Re\{a_{j}\left(t\right)\exp(i\phi_{j}\left(t\right))\}=Re\{a_{j}\left(t\right)\exp(i\int_{-\infty }^{t}\omega_{j}\left(t^{\prime }\right)dt^{\prime })\} \end{array}$$ (4)

where the *c* ^(*j*)^(*t*) represent the IMFs and *r*(*t*) the remaining non-oscillating trend. Furthermore, *a*_*j*_(*t*) denotes a time-dependent amplitude, *ϕ*_*j*_(*t*) = ∫*ω*_*j*_(*t*)*dt* represents a time-dependent phase and $\omega_{j}[rad/s]=\frac{d\phi_{j}(t)}{dt}$ denotes the related instantaneous frequency. Plotting both amplitude *a*_*j*_(*t*) and phase *ϕ*_*j*_(*t*) as a function of time for each extracted IMF represents a *Hilbert—Huang spectrogram* [34].

**Fig 2:**
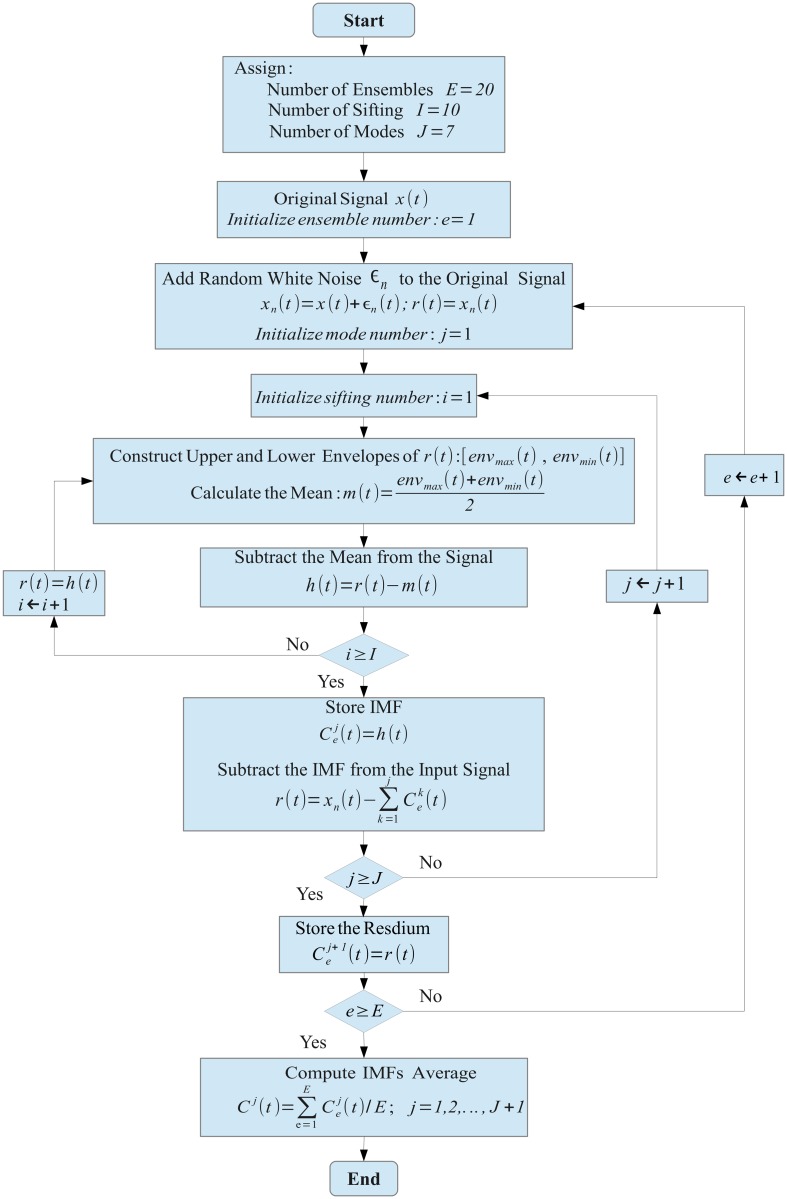
Flowchart diagram of the EEMD algorithm. The parameters were assigned according to values used in the numerical simulations.

During sifting, mode mixing as well as boundary artifacts can be avoided by a variant called *Ensemble Empirical Mode Decomposition* (EEMD) which has been introduced by [35]. It represents a noise-assisted data analysis method. Fig 2 illustrates EEMD data processing in a flowchart diagram. First white noise of finite amplitude is added to the data, and then the EMD algorithm is applied. This procedure is repeated many times, and the IMFs are calculated as an ensemble average, consisting of the signal and added white noise. With a growing ensemble number, the IMF converges to the true IMF [35]. Adding white noise to the data can be considered a physical experiment which is repeated many times. The added noise is treated as random noise, which appears in the measurement. In this case, the *n*–*th* noisy observation will be

$$\begin{array}{c} x_{n}\left(t\right)=x\left(t\right)+\epsilon_{n}\left(t\right)=\sum_{j}c_{n}^{\left(j\right)}\left(t\right)+r_{n}\left(t\right), \end{array}$$ (5)

where *x*(*t*) is the true signal, *ϵ*_*n*_(*t*) is the random noise and $c_{n}^{\left(j\right)}=c^{\left(j\right)}+\epsilon_{n}(t)$ represents the IMF obtained for the *n*-th noise observation. Therefore the resultant *IMF* *c* ^(*j*)^ is obtained by averaging all the $c_{n}^{\left(j\right)}$ of the ensemble. The flowchart, Fig 2, summarizes the main steps of the EEMD algorithm where the shifting process was implemented with a fixed number iterations instead of verifying the IMF condition [32]. Additionally, EEMD needs to prescribe two parameters, namely the size *E* of the ensemble and the standard deviation *σ*_*noise*_ of added Gaussian noise. Those two parameters were chosen empirically after several attempts which indicated an optimal ensemble number *E* = 20 and a proper standard deviation *σ*_*noise*_ = 0.2⋅*σ*_*signal*_, where *σ*_*signal*_ denotes the standard deviation of the signal amplitude distribution. Furthermore, because of the need to average corresponding IMFs over several trials, the number of IMFs into which the signals are decomposed has been fixed in advance. Empirically, after systematically varying the number of modes, a decomposition into 7 IMFs plus a residuum was considered most appropriate. Slightly abusing the definition of a residuum, by integrating the latter as an additional IMF, all signals were decomposed into 8 IMFs finally.

## Results

The following section will present results obtained from an EEMD analysis of EEG recordings of 18 subjects during a contour integration task. EEG data have been recorded simultaneously with fMRI scans. The evaluation of the latter has been reported recently by [27]. Results concerning raw data are presented at the level of event -related potentials (ERPs). Data is then decomposed with EEMD either using single trial recordings or ERPs.

### Illustrations of raw data characteristics

#### Grand average ERPs

**Fig 3:**
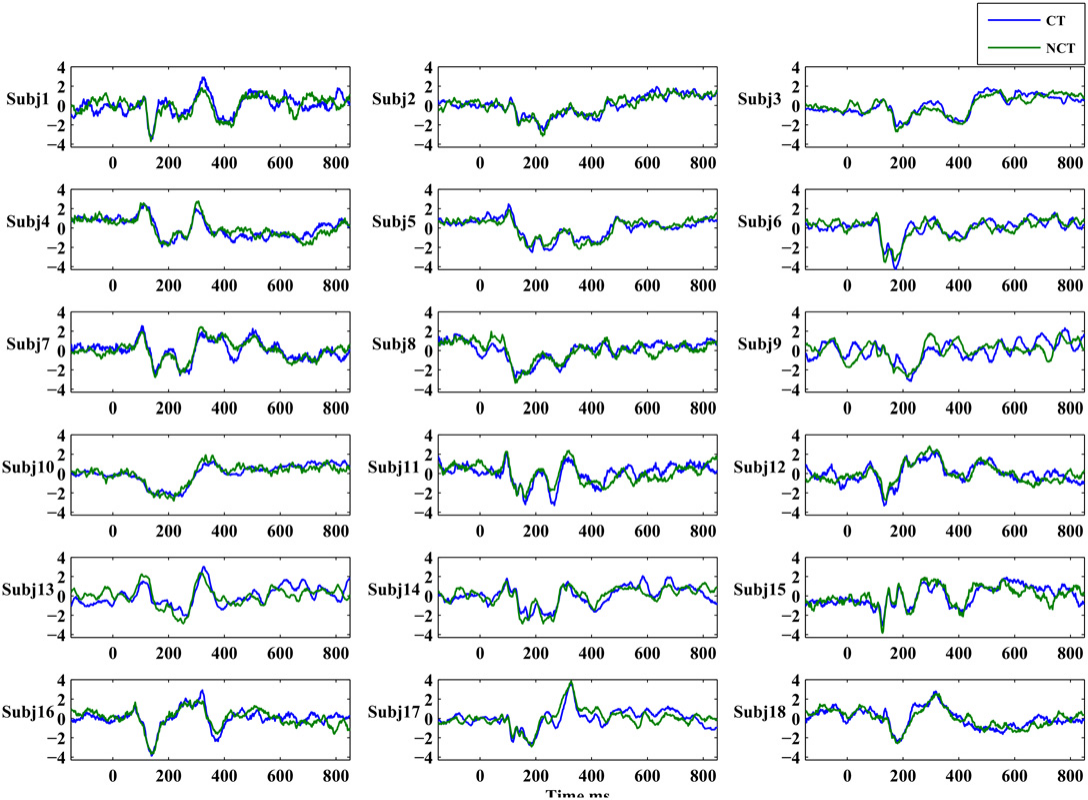
Global averages of individual ERPs elicited by the two stimulus conditions CT and NCT. The ERP amplitudes are normalized to zero mean and unitary standard deviation. ERPs for both conditions are superimposed onto each other, *blue*: Contour condition and *green*: Non-contour condition.

**Fig 4:**
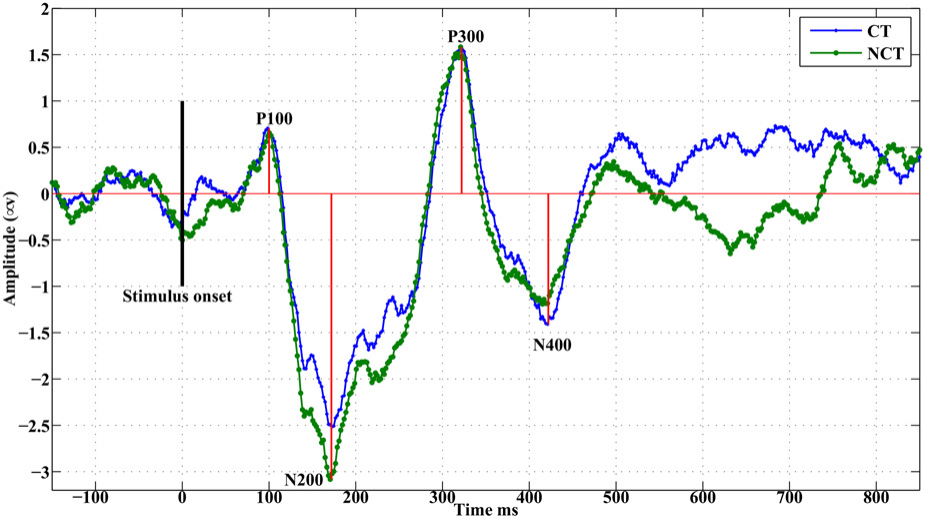
Grand average ERPs. They result from averaging individual global ERPs from 18 subjects.

Responses to Gabor stimuli exhibited a large biological variability as can be seen from Fig 3. There, for the purpose of illustration only, global ERPs are shown of all subjects as averages over all channels. Such global averages provide a rough idea about the extent of biological variability between individual stimulus responses. Fig 4 illustrates a typical global ERP response, obtained as a grand average over all channels and over all subjects. Such a grand average ERP exhibits four prominent ERP peaks after stimulus onset, which will be denoted according their latencies as *P*100, *N*200, *P*300 and *N*400, respectively [36]. As can be seen, typically one observes a positive peak occurring roughly at 100 [*ms*] (maximal response amplitude usually occurring between 70 [*ms*] and 180 [*ms*]) after stimulus onset, called *P*100. This is followed by a negative peak, called *N*200, with maximal amplitude between 150 [*ms*] and 260 [*ms*]). *P*300 identifies the next positive peak occurring usually between 270 [*ms*] and 370 [*ms*], followed by a late negative potential, denoted *N*400, at 370 [*ms*] to 450 [*ms*]. Note that the Gabor stimuli last for almost Δ*t*_*st*_ = 200 [*ms*] after stimulus onset *t*_0_, hence encompasses *P*100 and part of *N*200.

#### Individual ERPs

**Fig 5:**
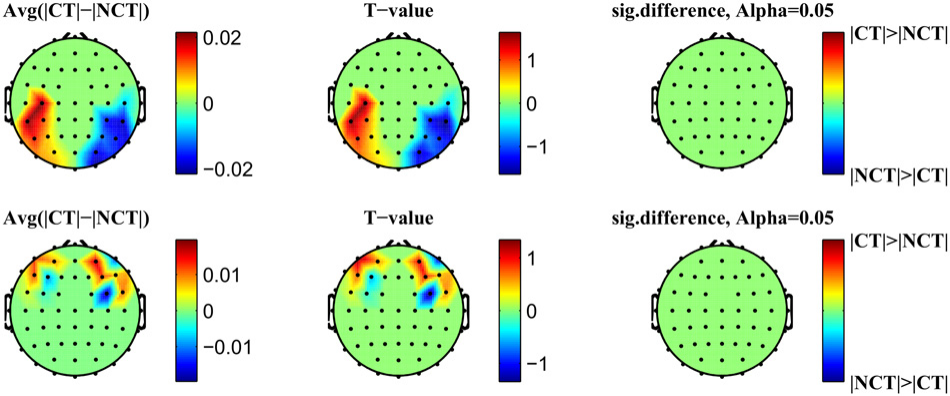
Head topography of the significant difference of the *P*100 signals. *Top*: Early response, *Bottom*: Late response

**Fig 6:**
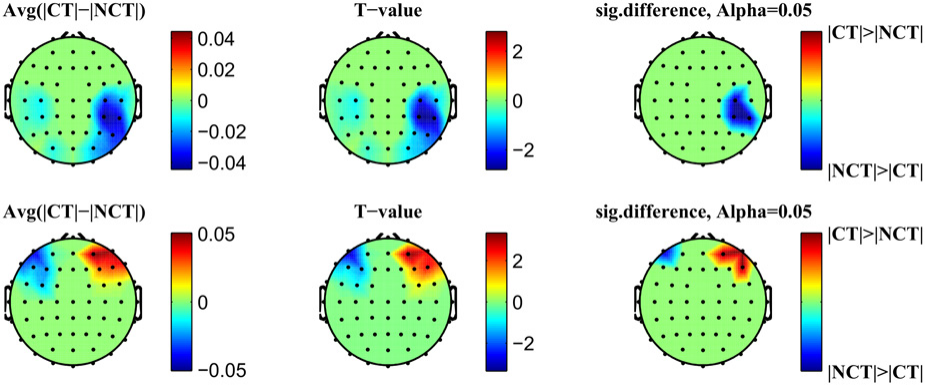
Head topography of the significant difference of the *N*200 signals. *Top*: Early response, *Bottom*: Late response

In the grand average ERP, differences between stimulus conditions mostly appear for the early responses, i. e. for *P*100 and most notably for *N*200, *N*400, and seemingly also between 600 [*ms*] and 800 [*ms*]. Mean amplitudes have been estimated therefore within intervals centered at these characteristic peaks. By visual inspection, it can be seen that for the ERP *P*100, in the occipital brain area an early response occurs during the time interval 60 [*ms*]–120 [*ms*] while in the frontal brain area the corresponding *P*100 peak shows up in the interval 120 [*ms*]–180 [*ms*]. Correspondingly, for the ERP *N*200 two intervals were considered corresponding to an early response at 150 [*ms*]–210 [*ms*] and a late response at 200 [*ms*]–260 [*ms*], respectively. Next, the difference of the absolute values of these mean response amplitudes, estimated at each channel and for each stimulus condition, has been computed. Finally, these differences have been averaged over the population of 18 subjects. A paired t-test identified the channels with the most significant differences in signal responses. The resulting values have been used to generate corresponding head topographies as illustrated in Figs 5 and 6. The topograms (see Fig 5) illustrate early and late response differences resulting from mean response amplitudes around the ERP *P*100. Obviously, early as well as late response differences appear to be insignificant at a confidence level of *α* = 0.05. On the contrary, early and late response differences estimated around the ERP *N*200 (see Fig 6) appear to be significant for the same confidence level. Early response differences are located in occipital areas of the brain, while late responses are found in frontal brain areas only. Additionally, such significant differences are detected only in the right hemisphere. Table 1 summarizes p—and T—values for significant early and late responses at various locations (channels) and for different confidence levels in case of ERP *N*200.

**Table 1:** Test statistics for ERP *N*200. Results of statistical tests of differences in mean ERP amplitudes for the event related potential *N*200 in early and late response areas. P- and T-values are reported for different significance levels: (*):*α* = 0.05, (**):*α* = 0.01, (***):*α* = 0.001

Early response			Late response		
p-value	T-value	Channel	p-value	T-value	Channel
0.042*	-2.199	C4	0.003**	3.417	AF4
0.015*	-2.720	CP6	0.022*	2.511	F6
0.012*	-2.830	CP4	0.005**	-3.255	AF7
			0.023*	2.500	AF8

### Event-related modes from EEMD decomposition

A more informative way to analyze ERPs concerns the application of signal decomposition techniques which offer a principled way of extracting characteristic features from the recordings which reveal significant response differences between both stimulus conditions. In this study, EEG signals are decomposed by applying ensemble empirical mode decomposition (EEMD). The latter will be applied to the set of signals in two different ways. Approach A preserves phase-locked modes and disregards non-phase-locked modes, while approach B considers both, phase-locked as well as non-phase-locked, modes during mode decomposition:

(A) After averaging over trials, EEMD is applied to all single channel ERPs, i. e. to $x_{avg}^{\left(ch\right)}(t_{n},CT)$ and $x_{avg}^{\left(ch\right)}(t_{n},NCT)$, respectively, thus yielding *event-related modes* (ERMs) which contain only those parts of the response signals that are phase-locked to the stimuli.
(B) Alternatively, EEMD is applied to each single trial response signal *x* ^(*ch*)^(*t*_*n*_, *s*) at each channel separately. Then all corresponding IMFs become, for each of the two stimulus conditions, averaged over all trials to yield related ERMs. The latter now also contain information about signal components non-phase-locked to the stimuli.

Before applying an EEMD decomposition, all signals were standardized to zero mean and unit variance (z-score).

#### Approach A: EEMD after averaging over trials

**Fig 7:**
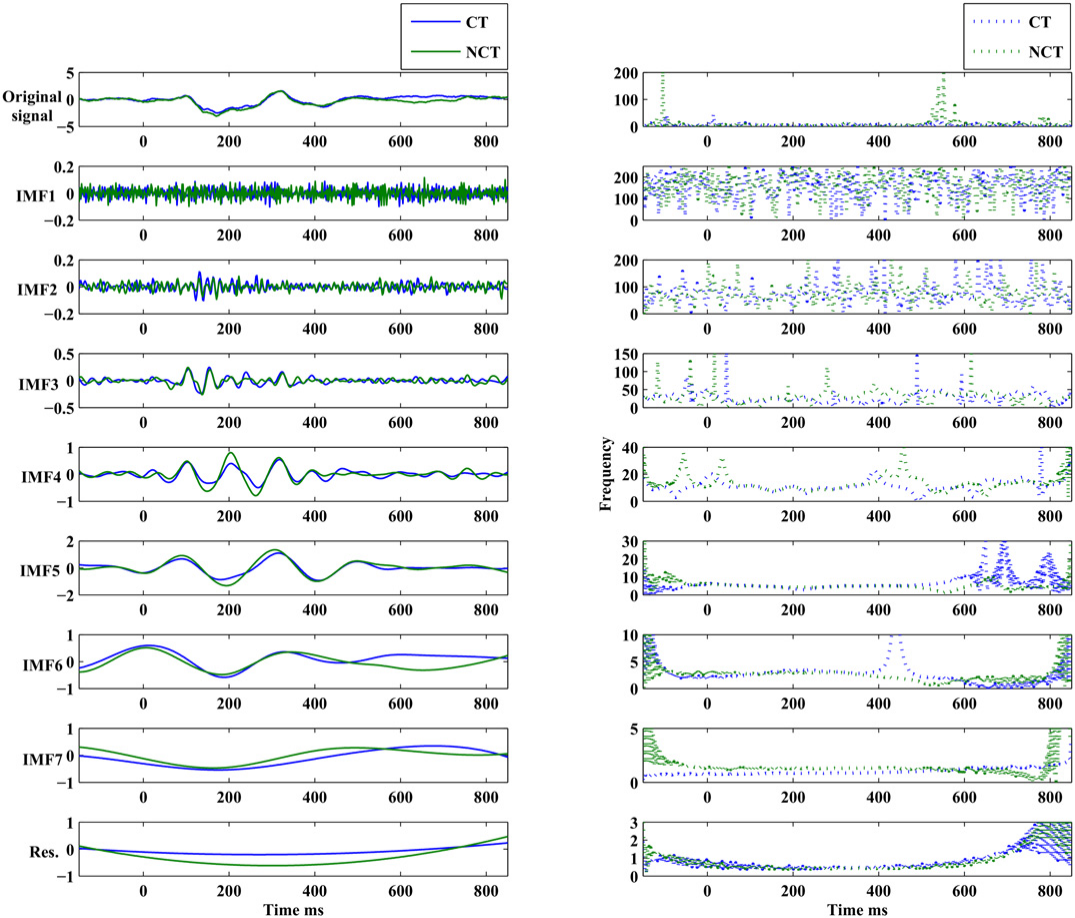
EEMD decomposition on grand average ERPs. Single subject ERPs were averaged over 18 subjects and are shown for two stimulus conditions: Contour and Non-contour. This signal is generated by averaging all signals from Fig 3 for the two conditions separately. Plots in the left column represent the averaged signal (on top) followed by its event-related intrinsic modes (ERMs). Plots in the right column present related Hilbert spectra for each of the corresponding ERMs in the left column.

The following Fig 7 illustrates ERMs, obtained from a grand average of global ERPs as shown in Fig 4, and their related Hilbert—Huang—Transforms. Especially *ERM*3–*ERM*6 show rather pronounced oscillations with stable frequencies (see the related Hilbert—Huang Transforms) ranging from 3 [*Hz*]–30 [*Hz*] roughly. Thus these oscillations represent characteristic EEG bands like theta—or alpha—bands.

Averaging has been performed for each stimulus condition separately and EEMD decomposition was performed for each ERP signal. Then IMF (ERM) amplitudes have been estimated within intervals centered at the characteristic peaks of the corresponding ERMs, i. e. for *P*100, *N*200 etc. as was explained above. Again, a subsequent paired t-test identified the channels with the most significant differences in signal responses. The resulting values have been used to generate corresponding head topographies. The latter are very similar to the ones obtained by approach B, but show lower significance levels. These results are summarized in Table 2 and Table 3. As can be seen from Table 2, early responses have been observed only for channels located in the occipital and parietal areas of the brain, while a highly significant late response has been observed for a channel in the frontal area of the brain. A similar observation holds for the entries of Table 3. Significant early response differences are more numerous for the different *N*200 ERM components, while again only a single frontal channel exhibited a significant response difference. Note that even the original ERP shows significant late response differences for the *N*200 ERP component.

**Table 2:** Approach A: Test statistics for *ERM*4 and *ERM*5 from *P*100. The table summarizes parameters of the test statistics (p-value, T-value) for *ERM*4 and *ERM*5 extracted from the ERP *P*100. An EEMD has been applied after averaging over trials to extract ERMs. P- and T-values are given for different confidence levels: (*):*α* = 0.05, (**):*α* = 0.01.

	Early response			Late response		
ERM	p-value	T-value	Channel	p-value	T-value	Channel
ERM 4	0.046*	-2.155	C6			
ERM 5	0.009**	-2.969	P8	0.002**	3.747	F4
	0.025*	-2.460	CP6			
	0.036*	-2.276	P6			

**Table 3:** Approach A: Test statistics for *ERM*5, *ERM*6 and *ERM*7 from *N*200. The table summarizes parameters of the test statistics for *ERM*5, *ERM*6 and *ERM*7 extracted from the *N*200 ERP. An EEMD has been applied after averaging over trials to extract ERMs. P- and T-values are given for different confidence levels: (*):*α* = 0.05, (**):*α* = 0.01.

	Early response			Late response		
ERM	p-value	T-value	Channel	p-value	T-value	Channel
ERM 5	0.010*	-2.884	P4	0.026*	2.438	F4
	0.008**	-3.034	P8			
	0.003**	-3.552	CP6			
	0.013*	-2.784	CP4			
	0.015*	-2.693	P6			
ERM 6	0.040*	-2.226	CP6			
	0.047*	-2.141	CP3			
ERM 7	0.008**	2.998	O1			
	0.002**	3.613	P7			

#### Approach B: EEMD before averaging over trials

Approach B consisted in performing an EEMD of all single trial signals separately. Then an average over trials has been performed for each IMF, each stimulus condition and each channel, resulting in corresponding ERMs. The further analysis estimating mean peak amplitudes of ERM components has been done in the same way as already discussed for approach A.

**Fig 8:**
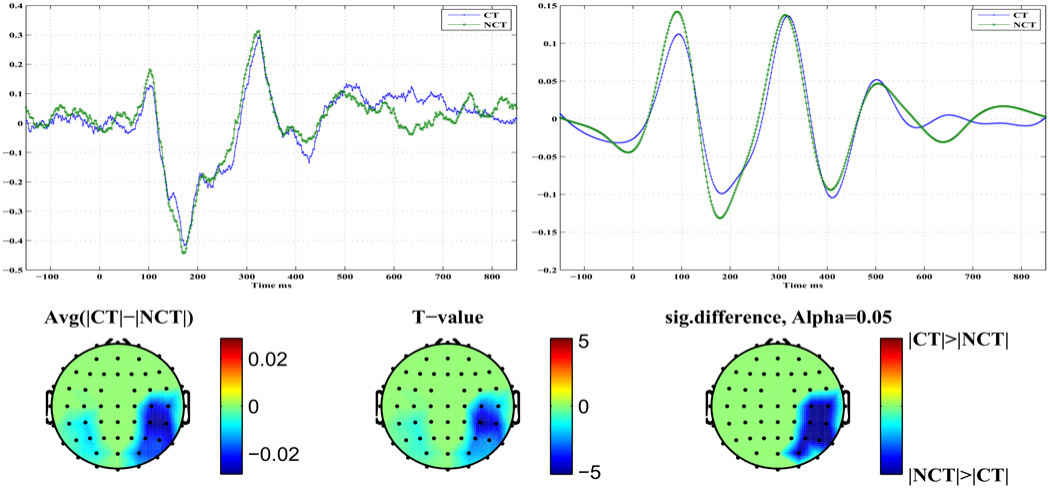
Early stimulus response at ERP *P*100. *Top Left*: Standardized original ERPs for both stimulus conditions. *Top Right*: *ERM*5 for both stimulus conditions. *Bottom*: Head topographies showing locations of significant differences in *ERM*5 amplitudes for both stimulus conditions.

Fig 8 illustrates differences in early *P*100 responses for both stimulus conditions. The top row presents on the right the component *ERM*5 for both stimulus conditions. It represents an average over all channels which exhibit a significant response difference at a confidence level of *α* = 0.05. On the left, the corresponding raw signal is presented for comparison. In the component *ERM*5, clear differences are seen in response amplitude for both stimulus conditions. In the bottom row, the topography resulting from differences in *ERM*5 amplitudes related to the ERP *P*100 at a significance level of *α* = 0.05 is shown. The rightmost topogram exhibits those brain regions where significant signal differences could be detected. As can be seen, early responses are localized in occipital and parietal areas of the right brain hemisphere only. There, the amplitude of the *P*100 component of *ERM*5 is larger for the stimulus condition NCT than for condition CT (see Fig 8). Furthermore, remember that for the raw *P*100 ERPs (Fig 8, top left) no statistically significant differences in response amplitude could be found. Note that the *P*100 peak maximum, both in the raw signal as well as in the *ERM*5, occurs shortly before *t* = 100 [*ms*], hence represents what we call an *early* stimulus response.

**Fig 9:**
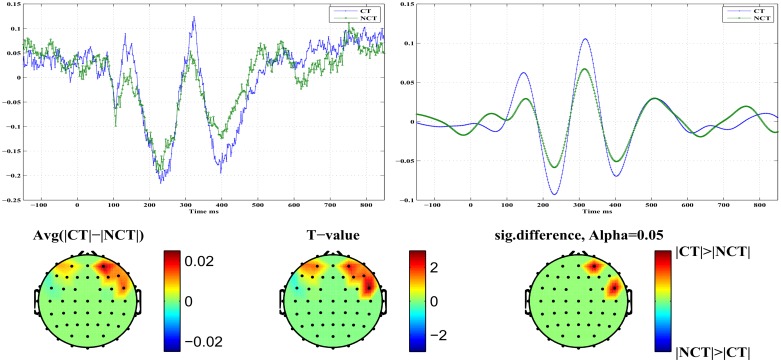
Late stimulus response at ERP *P*100. *Top Left*: Standardized original ERPs for both stimulus conditions. *Top Right*: *ERM*5 for both stimulus conditions. *Bottom*: Head topographies showing locations of significant differences in *ERM*5 amplitudes for both stimulus conditions.

Fig 9 illustrates differences in what we call *late* *P*100 responses for both stimulus conditions. The top row presents to the right the component *ERM*5 for both stimulus conditions. It represents an average over all channels which exhibit a significant response difference at a confidence level of *α* = 0.05. On the left, the corresponding raw signal is presented for comparison. In the component *ERM*5, the differences in response amplitude for both stimulus conditions are even more pronounced than in case of the early response. On the left, an average of the raw signals of all those channels which exhibit significant response differences is shown. In the bottom row the topography resulting from differences in *ERM*5 amplitudes measured for the ERP *P*100 at a significance level of *α* = 0.05. The rightmost topogram exhibits those brain regions where significant signal differences could be detected. As can be seen, late responses are localized in frontal and medio-temporal areas in the right hemisphere only. There the *ERM*5 amplitude for the stimulus condition CT is larger than for condition NCT (see Fig 9) contrary to what has been observed for the early *P*100 response in occipital areas. Note that the peak maximum occurs roughly at *t* ≃ 150 [*ms*]. Again for the raw *P*100 ERP signal no statistically significant differences between both stimulus conditions could be found. Table 4 summarizes, for approach B and the *P*100 ERP, parameters (p- and T-values) of the test statistics for *ERM*4 and *ERM*5 at different confidence levels. Note that an early response has been observed only for channels located in the occipital and parietal areas of the brain, while a late response is observed for a channel in the frontal area of the brain.

**Table 4:** Approach B: Test statistics for *ERM*4 and *ERM*5 from *P*100. The table summarizes parameters of the test statistics (p-value, T-value) for *ERM*4 and *ERM*5 extracted from the ERP *P*100. An EEMD has been applied *before* averaging over trials. P- and T-values are given for different confidence levels: (*):*α* = 0.05, (**):*α* = 0.01, (***):*α* = 0.001.

	Early response			Late response		
ERM	p-value	T-value	Channel	p-value	T-value	Channel
ERM 4	0.015*	-2.705	O2	0.025*	2.464	F3
	0.038*	-2.256	CP6	0.015*	2.695	F5
ERM 5	0.004**	-3.329	C4	0.006**	3.124	FC6
	0.009**	-2.902	P4	0.031*	2.354	AF4
	0.009**	-2.906	P8			
	0.000***	-4.384	CP6			
	0.000***	-5.311	CP4			
	0.031*	-2.345	PO4			
	0.049*	-2.123	C6			
	0.027*	-2.416	P6			

**Fig 10:**
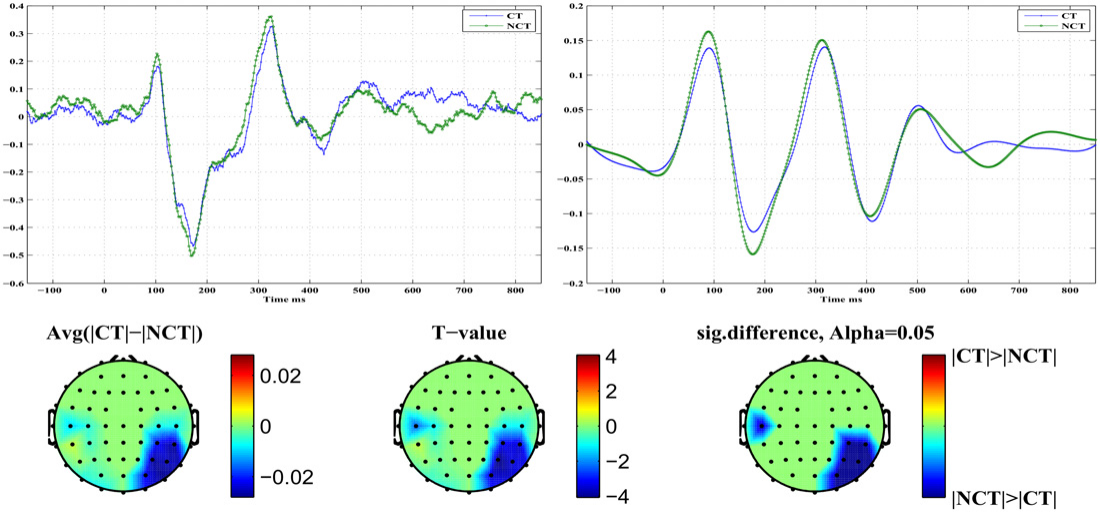
Early stimulus response at ERP *N*200. *Top Left*: Standardized original ERPs for both stimulus conditions. *Top Right*: *ERM*5 for both stimulus conditions. *Bottom*: Head topographies showing locations of significant differences in *ERM*5 amplitudes for both stimulus conditions.

Fig 10 illustrates differences in early *N*200 responses for both stimulus conditions. The top row presents on the right the component *ERM*5 for both stimulus conditions. It represents an average over all channels which exhibit a significant response difference at a confidence level of *α* = 0.05. On the left, the corresponding raw signal is presented for comparison. In the component *ERM*5, differences in response amplitude for both stimulus conditions are seen clearly. On the left, an average of the raw signals of all those channels which exhibit significant response differences is shown. The bottom row shows the topography resulting from differences in *ERM*5 amplitudes measured for the ERP *N*200 at a significance level of *α* = 0.05. The rightmost topogram exhibits those brain regions where significant signal differences could be detected. As can be seen, early responses are localized in occipital and parietal areas in the right hemisphere mainly but also in the left hemisphere, contrary to the *P*100 response. In the indicated brain areas, the *ERM*5 amplitude for the stimulus condition NCT is again larger than for condition CT (see Fig 10). Note that the negative peak occurs shortly before *t* = 200 [*ms*] and exhibits a clear shoulder shortly after *t* = 200 [*ms*].

**Fig 11:**
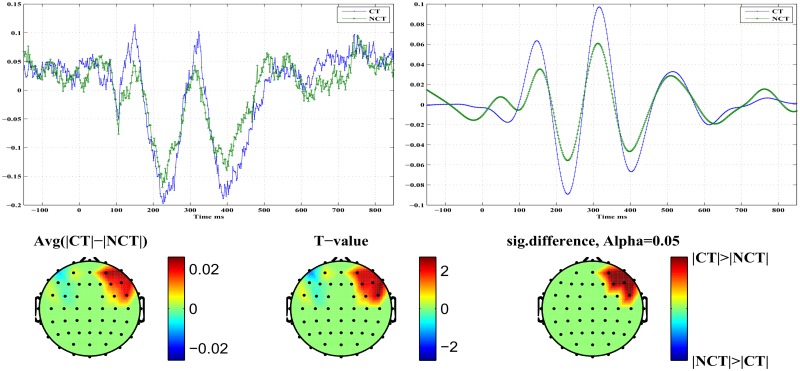
Late stimulus response at ERP *N*200. *Top Left*: Standardized original ERPs for both stimulus conditions. *Top Right*: *ERM*5 for both stimulus conditions. *Bottom*: Head topographies showing locations of significant differences in *ERM*5 amplitudes for both stimulus conditions.

Fig 11 illustrates differences in late *N*200 responses for both stimulus conditions. The top row presents on the right the component *ERM*5 for both stimulus conditions. It represents an average over all channels which exhibit a significant response difference at a confidence level of *α* = 0.05. On the left, the corresponding raw signal is presented for comparison. In the component *ERM*5, again very pronounced differences are seen in response amplitude for both stimulus conditions. The bottom row shows the topography resulting from differences in *ERM*5 amplitudes measured for the ERP *N*200 at a significance level of *α* = 0.05. The rightmost topogram exhibits those brain regions where significant signal differences could be detected. As can be seen, late responses are localized in frontal and medio-temporal areas in the right hemisphere only. There the *ERM*5 amplitude for the stimulus condition CT is again larger than for condition NCT. Note that for the late response the negative peak occurs shortly after *t* = 200 [*ms*]. Table 5 summarizes, for approach B and the *N*200 ERP component, parameters (p- and T-values) of the test statistics for *ERM*5–*ERM*7 at different confidence levels. Early responses were obtained again from channels in the occipital and parietal regions of the brain, while late responses were located at channels in frontal regions. Note that the difference between early and late responses is most clearly shown in *ERM*5.

**Table 5:** Approach B: Test statistics for *ERM*5, *ERM*6 and *ERM*7 from *N*200. The table summarizes parameters of the test statistics for *ERM*5, *ERM*6 and *ERM*7 extracted from the ERP *N*200. An EEMD has been applied *before* averaging over trials. P- and T-values are given for different confidence levels: *−*α* = 0.05, **−*α* = 0.01.

	Early response			Late response		
ERM	p-value	T-value	Channel	p-value	T-value	Channel
ERM 5	0.001***	-4.148	P4	0.040*	2.222	F4
	0.009**	-2.927	O2	0.014*	2.753	F8
	0.004**	-3.359	P8	0.015*	2.692	FC6
	0.003**	-3.470	CP6	0.039*	2.235	AF4
	0.005**	-3.225	CP4	0.048*	2.132	F6
	0.003**	-3.541	PO4	0.033*	2.329	AF8
	0.026*	-2.442	C5			
	0.003**	-3.440	P6			
	0.003**	-3.465	PO8			
ERM 6	0.032*	-2.339	C3	0.005**	-3.257	F8
	0.009**	-2.931	P7	0.008**	-2.991	FC4
	0.014*	-2.725	CP5			
	0.029*	-2.387	CP4			
	0.006**	-3.114	PO3			
	0.033*	-2.321	P5			
	0.036*	-2.275	P6			
	0.033*	-2.327	PO7			
ERM 7	0.030*	2.374	P3	0.042*	2.204	FC5
	0.010*	2.891	P4			
	0.014*	2.753	O1			
	0.046*	2.155	O2			
	0.014*	2.753	CP5			
	0.008**	2.981	PO3			
	0.022*	2.533	P5			
	0.002**	3.657	PO7			

The *ERM*5 is the mode that consistently has peaks with latencies similar to the studied ERP components. Concerning the component *P*100 of the mode *ERM*5, an early response is manifest on electrodes located in the occipital and parietal brain areas with a stronger response amplitude for the NCT—stimulus condition. On the contrary, a late *P*100 response is clearly detected in frontal brain areas with a larger response amplitude for the CT—stimulus condition. The delay of the late *P*100 response component amounts to Δ*t* ≈ 70 [*ms*]±10 [*ms*]. Furthermore, the response differences appear much more pronounced for the late *P*100 response compared to the early *P*100. A remarkable detail is seen in the *P*100 response for the NCT—stimulus condition. The *P*100 peak corresponding to the NCT—condition, which is visible in the early response (see Fig 8, top right), splits in the late response (see Fig 9, bottom right) into a double peak with one component at a time of occurrence of the early *P*100 response, and a later *P*100 response, peaking roughly 70 [*ms*] after the early response. This detail is only visible for the stimulus condition NCT and is absent for the CT—stimulus. The latter only shows the delayed component. Related head topographies indicate the brain areas where statistically significant response differences could be detected. They consistently indicate such differences only for the right hemisphere in broad agreement with recent findings about a right hemisphere specialization of contour integration [37]. They appear more focused in frontal areas while they are more diffuse and spatially extended in occipital, parietal and parieto-temporal areas for the early responses. Clearly, occipital as well as frontal areas are involved in contour integration as early as for the *P*100 response but most notably at the *N*200 level. This seems to support assertions of a top-down control in contour integration [18].

Similar results are obtained for the *N*200 response. An early *N*200 response prominently appears *before* *t* = 200 [*ms*], while a late *N*200 response is peaking *after* *t* = 200 [*ms*]. The response amplitude in the early phase is again larger, i. e. more negative, for the NCT—stimulus condition, while it is weaker in the late phase of the response. Once again, the response difference is more pronounced for the late response. Remarkably, the *N*200 response contour for the early CT—stimulus response exhibits a shoulder at the time where the late *N*200 response amplitude peaks. However, no clear double peak structure appears as for the *P*100 CT—stimulus response. If one compares the *ERM*5 signal structure related to the different electrodes located on a path from occipital to frontal a clear shift in the time of occurrence of the *N*200 peak from early to late is seen. Thus single *ERMs* allow to follow a precise timing of the *N*200 ERP along the visual processing pathway.

## Discussion

In the study we presented EEG recordings from 18 subjects which were participating in a perceptual learning task. More specifically, subjects were presented with Gabor stimuli which occasionally formed an open continuous contour. Subjects were asked to indicate the presence (CT) or absence (NCT) of such contours in their visual field at certain time points. After proper pre-processing, raw data have been averaged over all trials to extract event related potentials (ERPs) at every electrode. A global average of such ERPs is illustrated for all 18 subjects in Fig 3. Clearly, there is a large biological variability in the data with mostly little difference between both stimulus conditions. Note that data have been individually standardized to zero mean and unit variance. Relative differences between both stimulus conditions have been preserved that way. Fig 4 presents a grand average of individual global ERPs, indicating most clearly four prominent peaks named *P*100, *N*200, *P*300 and *N*400. This grand average suggests that differences in response amplitudes between both stimulus conditions are most probably to be expected at the early ERPs, i. e. at *P*100 and *N*200. But a statistical testing of observed differences in response amplitudes to both stimulus conditions could not proof any significant difference in the *P*100 ERP amplitudes at a significance level of *α* = 0.05 corresponding to *T* ≥ 2.1 (see Fig 5). For the *N*200 ERP response amplitudes, at the same significance level, statistically significant differences could be found at several electrodes mainly located in the occipital and parietal areas of the brain but also in frontal brain areas. Interestingly, response amplitudes peaked *before* *t* = 200 [*ms*] in the occipital and parietal areas but showed a late response *after* *t* = 200 [*ms*] for the frontal electrodes. Henceforth, these response are called early and late responses, respectively. Early and late responses show a delay of roughly Δ*t* = 100 [*ms*] and correlate well with electrode locations in either the occipital or frontal brain areas.

This rough analysis of raw ERP signals has been improved by applying a signal decomposition technique based on an ensemble empirical mode decomposition (EEMD) as proposed by Huang et al. [26, 35, 38]. The analysis has been performed in two different ways: either EEMD has been applied *before* or *after* averaging over trials. The resulting component signals extracted have been called event related modes (ERMs). EEMD before averaging delivered better results with respect to statistical measures, probably because of unfavorable signal compensations through averaging. Hence, only these results have been presented in detail. For illustrative purposes only, Fig 7 presents a grand average EEG signal extending from 150 [*ms*] before stimulus onset at *t*_0_ = 0 to 850 [*ms*] after *t*_0_. While the original signals exhibit only small differences, mainly around ERP *N*200, some of the extracted ERMs indicate clear differences between both stimulus conditions. The related Hilbert—Huang spectra indicate decent regularities in these ERMs with characteristic frequencies which stay largely constant over the time span considered. Most notable differences are seen for *ERM*3, *ERM*4, *ERM*5 and *ERM*6. For example, *ERM*3 shows a dominant frequency around *ν* = 25 [*Hz*], corresponding to the *β*—band, *ERM*4 shows oscillations around *ν* = 10 [*Hz*] which represents an *α*—activity, *ERM*5 oscillates with roughly *ν* = 5 [*Hz*] indicating a *θ*—wave and, finally, *ERM*6 is dominated by an oscillation with *ν* ≤ 2 [*Hz*] corresponding to a *δ*—wave. *ERM*2 is indicative of some high frequency (*ν* ≥ 30 [*Hz*]) activity which might contain *γ*—wave activities. Note that these spectral characteristics agree between both stimulus conditions.

**Fig 12:**
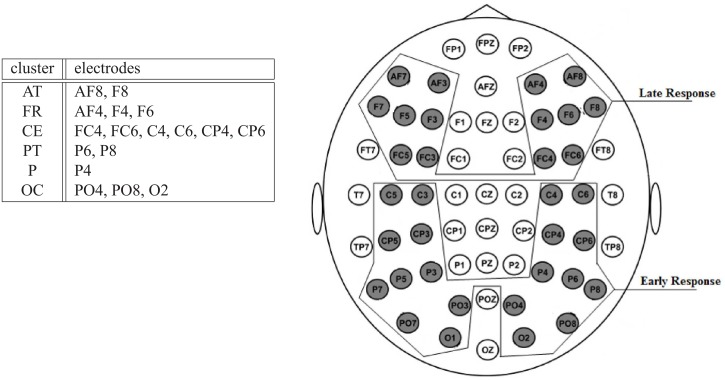
Pooling of electrodes into 6 clusters and the electrocap layout. *Left*: Notation refers to the right hemisphere. The left hemisphere is organized similarly with the electrodes with odd number.*Right*: It shows the two groups of electrodes related with early and late responses

Even further insight is provided from ERMs resulting from an EEMD application to the single trial signals. The resulting intrinsic mode functions (IMFs) have then been averaged over all trials to result in corresponding ERMs. The largest differences between stimulus conditions are seen for *ERM*4–*ERM*6, i. e. in the *α*−, *θ*− and *δ*—bands, around *P*100 and *N*200. A general characteristic of the ERP components is that a clear transition from an early response to a late response is observable which exhibits a strong correlation to a spatial representation of the stimulus response either in the occipital or the frontal brain areas. Central brain areas show signs of both, early and late responses. The electrodes, where either the early or the late response could be observed, are shown in Fig 12.

**Fig 13:**
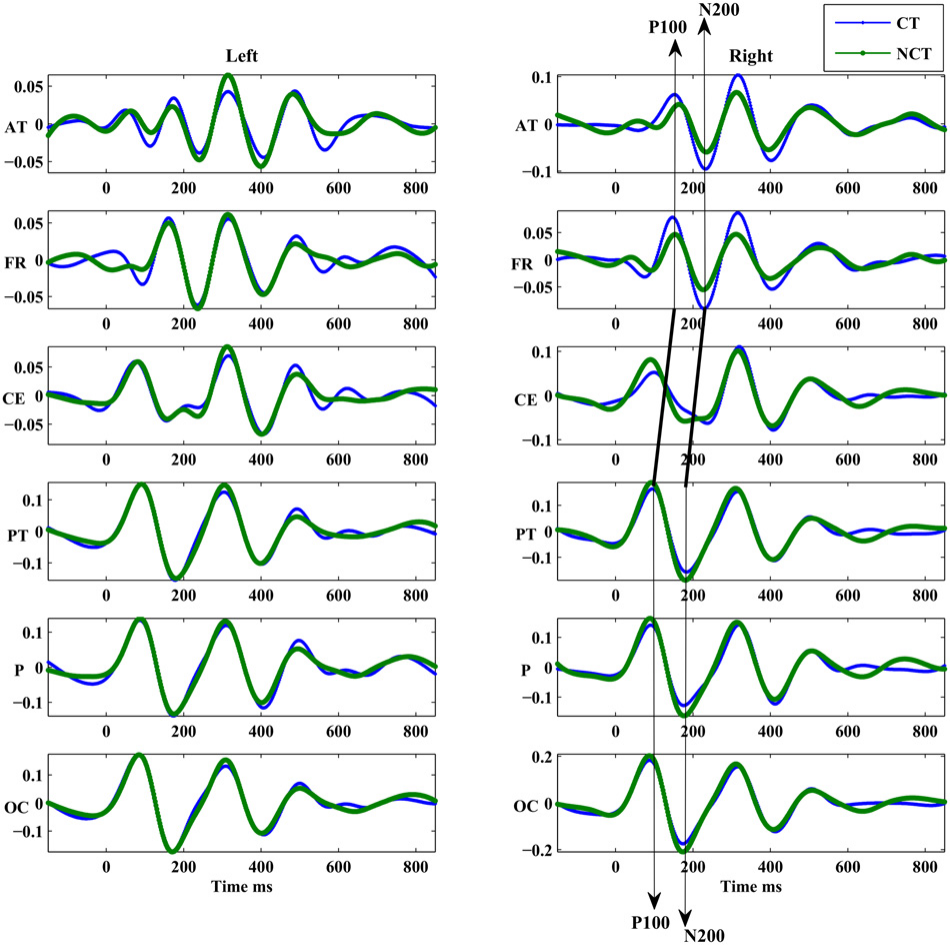
The pooled *ERM*5 responses. The *ERM*5 are averaged in pools of electrodes located on different regions.

While studying the switching between early and late responses in more detail, signals have been pooled according to a clustering scheme proposed by [39] which divides each hemisphere into six electrode clusters. The grouping of electrodes on the right hemisphere is given in table of the Fig 12. The difference on the latency of the peaks of *ERM*5 related with the ERP components is clearly visible on the ERM averages of the pooled electrodes. Fig 13 illustrates that those peaks show earlier in the parietal -occipital regions (*OC*, *P*, *PT*) and later on the frontal (*FR*, *AT*). The differences in latencies of *ERM*5, corresponding to the pooled signals of the right hemisphere, are marked in Fig 13 by the straight lines. Similar differences are observed in the left hemisphere though supported by a smaller statistical significance between the conditiond (see Tables 4 and 5). However, the *ERM*5 of the CE cluster clearly exhibits a double peak structure of the *N*200 ERP component showing early and late response components simultaneously. This is justified by the average of the two kind of signals, e.g, early and late responses. Surprisingly, the pooled *ERM*5 of the frontal region shows a similar double peak structure for the ERP component *P*100.

Because the human head is a volume conductor, the peaks in the ERP and ERM amplitudes observed at frontal and occipital electrodes do not necessarily imply that the brain activity was generated within frontal and occipital sources, respectively. However, a change in topography as seen from the earlier to the later phases of the *P*100 and *N*200 peaks clearly indicates different distributions of neural activity in each phase [40, 41]. Thus, the data favor the view that contour processing involves activity within distributed brain networks, rather than focal activity within the lower visual cortex [42]. This notion is broadly compatible with results from recent EEG or combined EEG and fMRI investigations [16, 17, 22]. On contrast to previous studies, we used a data-driven approach for EEG signal decomposition. The findings thus give an independent proof that contour integration depends on distributed network activity.

The time and frequency ranges where differences between stimulus conditions occurred are comparable to those of previous studies on oscillatory brain responses during contour integration. We found the strongest differences at parietal and occipital sites occurring < 200*ms* after stimulus onset and in low-to-mid frequencies [16, 18, 21]. A prominent role for beta oscillations in contour integration, as observed in our previous studies, did not show up in the data. Note, however, that a transient increase in beta power would not necessarily lead to increased amplitudes within one specific ERM. An ERM-based analysis or the EEG is thus not well-suited for validating the results of previous Fourier-based analyses.

In this study we used exclusively Gabor arrays as visual stimuli. It could thus be objected that the results are not specific for contour integration, but reflect some general visual processes under conditions of low stimulus visibility. Indeed, a recent fMRI study revealed that brain activity in visual as well a prefrontal areas increased with the presentation duration and so with the visibility of shortly presented gratings [43]. The role of the frontal cortex in this and in our study is yet unclear. Previous authors argue that frontal cortical activity is related to a conscious visual experience or to post-perceptual processes like motor preparation [44, 45]. On the other hand, the frontal cortex can also be directly involved into the actual stimulus processing [43]. The fact that posterior and frontal activity differences between contour and non-contour conditions occurred very early during processing is more in line with the latter interpretation. Thus, it seems warranted to conclude that the observed differences in brain activity originate from contour processing per se.

## Conclusion

In this investigation we used a data-driven approach to analyze EEG time series recorded during a contour integration task employing Gabor stimulus patterns. An EEMD analysis allows to extract component signals which show differences between stimulus conditions with clear statistical significance. Most notably *ERM*5 exhibits very pronounced differences between contour and non-contour stimulus responses. From an approach employing pooled electrode signals, latencies between early and late responses can be identified and quantified whereby early responses are registered at electrodes in the posterior part of the scalp, while late responses are visible only in the frontal part of the scalp. The pool of electrodes in the central part of the brain even exhibits both early and late responses in pronounced double peak structures. Together these findings provide independent evidence of a view of extended neuronal networks involved in visual processing, especially contour integration. Future work will concentrate on identifying related sources of neuronal activation via inverse modeling. The software tools developed within this study have been integrated into a toolbox *EMDLAB* which will be provided as a plug-in to the *EEGLAB* toolbox available at http://sccn.ucsd.edu/eeglab/.
